# Changes in B-type natriuretic peptide and related hemodynamic parameters following a fluid challenge in patients with severe sepsis or septic shock

**DOI:** 10.1186/cc10838

**Published:** 2012-03-20

**Authors:** S Omar, LR Mathivha, A Ali

**Affiliations:** 1 Wits University, Johannesburg, South Africa

## Introduction

The aim of the study is to describe the hemodynamic changes and relate them to the changes in B-type natriuretic peptide (BNP) following fluid challenge in patients with severe sepsis and septic shock.

## Methods

This prospective observational study enrolled 30 patients with severe sepsis or septic shock who required a fluid challenge within 48 hours of admission to the ICU. All patients had a basic cardiac echocardiogram (echo) performed, blood for BNP collected and baseline hemodynamic measurements recorded. A 500 ml colloid challenge was administered within 30 minutes. The echo and hemodynamic measurements were repeated at this point. One hour after the fluid challenge the BNP test was repeated.

## Results

One patient was excluded due to missing data. There were significant increases in mean arterial pressure (MAP), left ventricular dimensions at the end of diastole and systole, stroke volume (SV), cardiac output (CO) and BNP after the fluid challenge, while the heart rate decreased. Impaired cardiac contractility was defined as an ejection fraction (EF) <50%. The left ventricular end-systolic dimension (LVESd) before (4 cm vs. 2.9 cm) and after the fluid challenge (4.2 cm vs. 3.29 cm) was significantly greater (statistically and beyond reference intervals) in the EF <50% group compared to the EF >50% group. In the group with EF <50, the median LVEDd2 and LVESd2 post fluid challenge increased to values of 5.72 cm and 4.28 cm respectively (above the reference thresholds). For the group with EF >50, the median post-challenge LVEDd2 and LVESd2 increased significantly (*P *= 0.01) to 5.38 cm and 3.39 cm but within the reference thresholds. The BNP increased by 53.6% in the EF <50 group in contrast to a decrease by 12.7% in the EF >50 group. The median EF in the EF <50 and EF >50 groups were significantly different (0.44 vs. 0.66 respectively). Multiple regression analysis found LV dimension at end diastole at baseline was one of four independent predictors of an increase in %BNP.

## Conclusion

A significant increase in %BNP after a fluid challenge (irrespective of initial value) may indicate that cardiac contractility is impaired and the LV dilated, indicating a strategy away from fluid resuscitation and towards inotrope use.
